# Visualization of Resected Area in Endonasal Endoscopic Approach versus Transcranial Approach for Skull Base Meningiomas by Voxel-Based-Lesion Mapping

**DOI:** 10.3390/brainsci12070875

**Published:** 2022-06-30

**Authors:** Hiroshi Uda, Takehiro Uda, Manabu Kinoshita, Haruhiko Kishima, Yuta Tanoue, Atsufumi Nagahama, Toshiyuki Kawashima, Hiroki Ohata, Kosuke Nakajo, Hiroki Morisako, Takeo Goto

**Affiliations:** 1 Department of Neurosurgery, Graduate School of Medicine, Osaka Metropolitan University, Osaka 545-8585, Japan; udah.1234@icloud.com (H.U.); yuta_tanoue_0712@yahoo.co.jp (Y.T.); naga.atsu0327@gmail.com (A.N.); toshiyuki1986.331.24ser@gmail.com (T.K.); casino_drive810@yahoo.co.jp (H.O.); kousuke19841984@yahoo.co.jp (K.N.); hmorisako@med.osaka-cu.ac.jp (H.M.); gotot@med.osaka-cu.ac.jp (T.G.); 2 Department of Neurosurgery, Graduate School of Medicine, Osaka University, Suita 565-0871, Japan; mail@manabukinoshita.com (M.K.); hkishima@nsurg.med.osaka-u.ac.jp (H.K.); 3 Department of Neurosurgery, Asahikawa Medical University, Asahikawa 078-8510, Japan

**Keywords:** skull base meningioma, endonasal endoscopic approach, voxel-based-lesion mapping

## Abstract

Background: We aimed to evaluate the resected area of endonasal endoscopic approach (EEA) and transcranial approach (TCA) for skull base meningiomas (SBMs) using voxel-based-lesion mapping and visualized the appropriate tumor location in each approach. Methods: We retrospectively examined 182 patients with SBMs who underwent tumor resection in our hospital between 2014 and 2019. Pre- and post-operative SBMs were manually delineated on MRI to create the voxels-of-interest (VOI_pre_ and VOI_post_) and were registered onto the normalized brain (normalized VOI_pre_ and normalized VOI_post_). The resected map was created by subtracting normalized VOI_post_ from the normalized VOI_pre_ divided by the number of cases. The resected maps of TCA and EEA were compared by subtracting them. Results: Twenty patients underwent EEA and 135 patients underwent TCA. The tumor resected map demonstrated that the resected area of EEA frequently accumulated on the central skull base, while that of TCA accumulated near the central skull base. The border of both approaches matched the circle that connects neural foramens at the skull base. Conclusions: The resected area of SBMs by EEA and TCA was well visualized by voxel-based-lesion mapping. The circle connecting the neural foramens was the border of EEA and TCA.

## 1. Introduction

Surgical treatment for skull base meningiomas (SBMs) aims to achieve radical resection while preserving neurological functionality. It is challenging to approach medially seated SBMs via transcranial approach (TCA), as the operative fields are deep-seated and surrounded by critical neurovascular structures. The endonasal endoscopic approach (EEA) has also been recently applied for central SBMs [1]. The critical advantage of EEA is that it enables direct access to the tumor’s attachment from the medial side of the SBM while avoiding critical neurovascular structures. Controversy exists around whether EEA or TCA is the preferred choice for treatment of SBMs [2,3,4]. The resectability of SBMs has been analyzed in previous studies using surgeons’ impressions of the resection rates [5,6].

Voxel-based-lesion mapping on MRI allows quantitative visualization of the therapeutic effect of brain tumors [7]; therefore, the authors of this study aimed to objectively evaluate resected areas and visualize the suitable location in each approach of EEA and TCA for surgical resection of SBM.

## 2. Materials and Methods

### 2.1. Patients

We retrospectively examined 182 consecutive surgeries for patients with SBMs, who underwent tumor resection in our hospital between January 2014 and December 2019. Among them, we excluded two cases with radiation-induced meningiomas, four with multiple lesions, 18 with World Health Organization (WHO) grade 2 or 3 meningiomas, and three cases who did not undergo enhanced magnetic resonance imaging (MRI). The remaining 155 surgeries were included in our cohort. The institutional ethics committee approved this study. Written informed consent for the publication of images and clinical data was obtained from all patients.

### 2.2. Tumor Location and Surgical Strategies

SBMs were classified into four subgroups according to the location of the tumor: frontal base (FB), sphenoid-middle fossa (SpM), petroclival (PC), and posterior fossa (PF) [8] The routes utilized for TCA were the front basal route for FB, the orbitozygomatic route for SpM, the transpetrosal route for PC, and the suboccipital route for PF. We selected EEA for central SBMs that were difficult to approach by TCA.

EEA and TCA were considered separate surgeries in cases of combined surgeries for large SBMs with broad attachments. Tumors adherent to critical neurovascular structures were retained and scheduled stereotactic radiosurgery (SRS) was performed.

### 2.3. Evaluation of Clinical Information

All clinical data, including neurological findings, operative records, and radiological images, were obtained from the medical records. All patients were followed up for more than three months after the surgery. Clinical outcomes were evaluated by comparing preoperative Karnofsky performance status (KPS) scores and three months postoperatively. We defined an increase in KPS score as an improvement of clinical outcomes. Neurological deficits were also evaluated three months after the surgery. A recurrence was defined as a tumor enlargement which required additional surgeries or radiation therapy. Cerebrospinal fluid (CSF) leakage was defined as a complication when the surgical repair was needed.

### 2.4. Evaluation of Tumor Removal Rates by Intraoperative Findings

We evaluated tumor removal rates based on intraoperative findings, a method in-line with conventional methods of assessment; gross total resection (GTR) was defined as resection without microscopic remnants, subtotal resection (STR) was defined as resection of over 95% of the tumor, and partial resection (PR) was defined as resection of under 95% of the tumor.

### 2.5. Voxel-Based-Lesion Mapping

**Figure 1:**
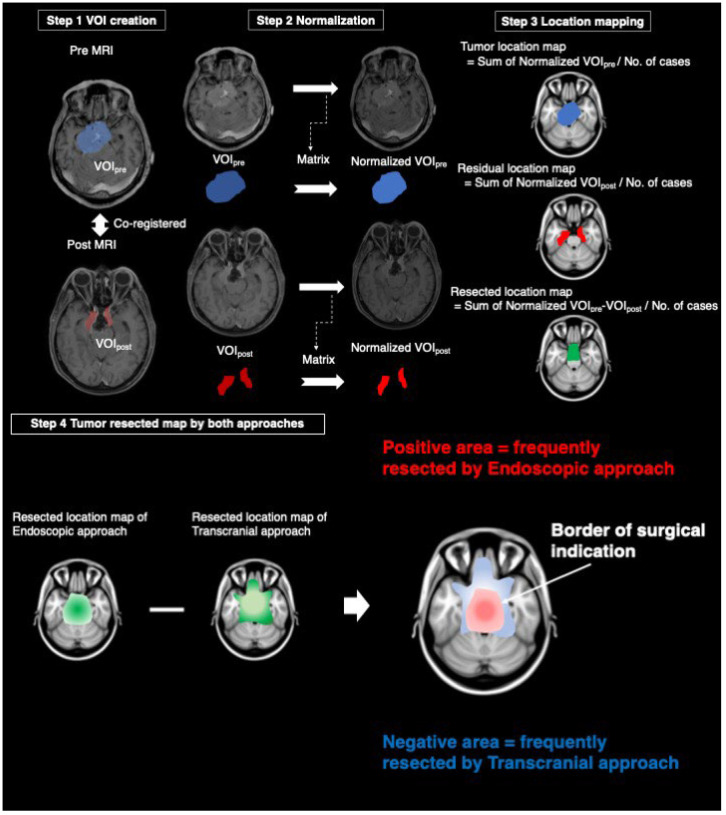
The workflow of voxel-based-lesion mapping. Step 1: The creation of the voxels-of-interest (VOIs) from pre-and post-MRI after co-registration. Step 2: Reslicing of VOIs using a matrix obtained from the transformation of pre-and post- magnetic resonance images into normal brain. Step 3: Tumor and residual location maps were created by the sum of normalized VOI_pre_ and normalized VOI_post_ divided by a number of cases, respectively. Resected location map was created by the subtraction of the residual location map from the tumor location map divided by a number of cases. Step 4: Tumor resected maps by both approaches were obtained by subtracting the resected location map of transcranial approach from that of endoscopic approach. VOI: the voxels-of-interest, MRI: magnetic resonance images.

All imaging data were acquired from gadolinium-enhanced T1-weighted images of either 1.5 or 3.0T MRI. Voxel-based lesion mapping was performed using an image analysis software developed in-house in combination with the Oxford Centre for Functional MRI of the Brain (FMRIB) Linear Image Registration Tool (FLIRT) provided by FMRIB Software Library (FSL) [9]. The in-house software was developed using Matlab R2017A (Mathworks, Natick, MA, USA), and seamless data transfer was carried out between Matlab-based in-house software and FSL via FSL integration into Matlab [7,10] All Digital Imaging and Communications in Medicine format images were first converted to the Neuroimaging Informatics Technology Initiative (NIFTI) format using Mango ver. 4.1 (University of Texas Health Science Center: http://rii.uthscsa.edu/mango/mango.html (accessed on 2 April 2019) [11]. The workflow of voxel-based-lesion mapping was shown in Figure 1. Step 1: Pre- and post-operative images in NIFTI formats were co-registered by VINCI ver. 4.92 (Max Planck Institute for Metabolism Research: http://vinci.sf.mpg.de/ (accessed on 1 August 2018) [12]. Pre- and post-operative SBMs were delineated by manually tracing performed by experienced surgical neuro-oncologists (H.U. and M.K.) to create the voxels-of-interest (VOIs). VOIs obtained from pre-and post-operative images were named “VOI_pre_” and “VOI_post_”, respectively. Step 2: Pre- and post-operative MRI were normalized onto a 1.0 mm isotropic, high-resolution T1-weighted brain atlas, provided by the Montreal Neurological Institute (MNI) 152 using a mutual information algorithm with 12 degrees of freedom transformation with FSL-FLIRT. By using the obtained transformation matrices as normalized VOI_pre_ and normalized VOI_post_, VOI_pre_ and VOI_post_ were then resliced and registered onto MNI152. These procedures were necessary to perform the lesion mapping of VOIs in standard MNI152 space. Overall, we collected normalized VOI_pre_ and normalized VOI_post_ from 155 surgeries. Step 3: We created a tumor location map from the sum of normalized VOI_pre_ divided by the number of cases and a residual location map from the sum of normalized VOI_post_ divided by the number of cases. The resected location map was created by subtracting the sum of normalized VOI_post_ from the sum of normalized VOI_pre_ divided by the number of cases. Step 4: A tumor resected map by both approaches was obtained by subtracting the resected location map of TCA from that of EEA. Areas with positive values indicated a frequently resected area by EEA, and areas with negative values indicated a frequently resected area by TCA. The border of the positive and negative areas suggests the border of surgical indication of both approaches.

## 3. Results

### 3.1. Patient Characteristics

A total of 20 EEA surgeries and 135 TCA surgeries were performed. The EEA group consisted of 7 males and 13 females, with a mean age of 57.8 ± 11.8 SD. The TCA group consisted of 36 males and 99 females with a mean age of 57.8 ± 12.2 SD. The location of EEA comprised three surgeries (15.0%) with FB, ten surgeries (50.0%) with SpM, and seven surgeries (35.0%) with PC. The TCA group consisted of 31 surgeries (23.0%) with FB, 36 surgeries (26.7%) with SpM, 46 surgeries (34.1%) with PC, and 22 surgeries (16.3%) with PF. Staged-combined surgery of EEA and TCA was performed in eight surgeries for four patients. The characteristics of patients was shown in Table 1.

### 3.2. Microscopic Tumor Removal Rates

We achieved GTR in zero surgeries (0%), STR in 17 surgeries (85.0%), and PR in three surgeries (15.0%) in the EEA group. We achieved GTR in 60 surgeries (44.4%), STR in 63 surgeries (46.7%), and PR in 12 surgeries (8.9%) among the patients in the TCA group.

### 3.3. Recurrence Rates and Adjuvant Therapies

Five patients (25.0%) experienced recurrence from residual tumors after EEA: a patient with cavernous meningioma underwent an adjuvant surgery and two SRSs; another patient with cavernous meningioma underwent an adjuvant surgery and SRS; two patients of petroclival meningioma underwent an adjuvant surgery; and a patient of parasellar meningioma underwent an adjuvant SRS. Twenty-one patients (15.6%) experienced a recurrence of tumor after TCA; a patient with optic canal meningioma underwent an adjuvant SRS; two patients of tuberculum sellae meningioma underwent an adjuvant surgery; seven patients with petroclival meningioma underwent an adjuvant SRS; two patients with petroclival meningioma underwent an adjuvant SRS and an adjuvant surgery; a petroclival meningioma patient underwent an adjuvant SRS and two adjuvant surgeries; another patient of petroclival meningioma underwent two adjuvant SRSs and an adjuvant surgery; a patient of cavernous meningioma underwent an adjuvant SRS; two other patients with cavernous meningioma underwent an adjuvant surgery; a patient of sphenoid ridge meningioma underwent an adjuvant surgery; and two patients with sphenoid ridge meningioma underwent an adjuvant SRS. Twelve patients (8.9%) after TCA and one patient (5.0%) after EEA underwent scheduled SRSs of the remaining tumors.

### 3.4. Clinical Outcomes by KPS Scoring

In total, 1 patient (5.0%) improved, and 19 patients (95.0%) displayed no change in KPS score after EEA. A total of 19 patients (14.1%) improved, 104 patients (77.0%) demonstrated no change, and 12 patients (8.9%) demonstrated deterioration in KPS score after TCA.

### 3.5. Permanent Neurological Deficits

One patient (5.0%) suffered from diplopia after EEA. A total of 3 patients (2.2%) suffered from anosmia, 3 patients (2.2%) had a visual defect, 19 patients (14.1%) suffered from diplopia, 11 patients (8.1%) suffered from facial numbness, 3 patients (2.2%) suffered from facial palsy, 2 patients (1.5%) suffered from hearing loss, 7 patients (5.2%) suffered from dysphagia, 5 patients (3.7%) suffered from motor paralysis, and 1 patient (0.7%) suffered from ataxia after TCA. One patient who underwent TCA suffered from aspiration pneumonia and died during the perioperative period.

### 3.6. CSF Leakage

Postoperative CSF leakage occurred in three surgeries (15.0%) of EEA and two surgeries (1.5%) of TCA. All patients recovered after the surgical repair.

### 3.7. Tumor Resected Map by Both Approaches

**Figure 2:**
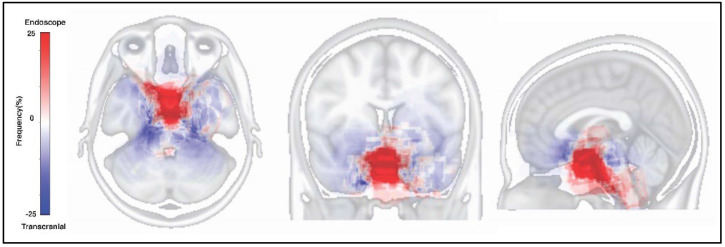
Tumor resected map on the Montreal Neurological Institute 152 standard brain atlas. The red area indicates the frequently resected area by endonasal endoscopic approach. The blue area indicates the frequently resected area by transcranial approach.

**Figure 3:**
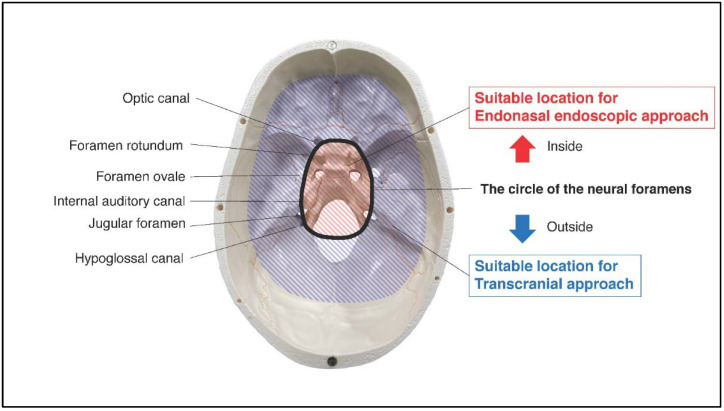
Red-striped region indicates the area with frequently resected by endonasal endoscopic approach. Blue-striped region indicates the area with frequently resected by transcranial approach. The border of them matched well the circle of the neural foramens (optic canal, foramen rotundum, foramen ovale, internal auditory canal, jugular foramen, and hypoglossal canal).

The tumor resected map by both approaches for SBMs is shown in Figure 2. The resected location map of EEA frequently accumulated on the central skull base, while that of TCA accumulated around the central skull base. The border of both areas matched the circle which connects the skull base neural foramens, including optic canal, foramen rotundum, foramen ovale, internal auditory canal, jugular foramen, and hypoglossal canal (Figure 3). The resected area by EEA and TCA covered all areas of the skull base. We have provided a Supplementary Dataset of tumor location maps, residual location maps, and resected location maps.

## 4. Discussion

### 4.1. Summary of This Study

We retrospectively studied 155 consecutive surgeries in patients who underwent tumor resection for SBMs. Voxel-based-lesion mapping was used to visualize the resected area of SBMs by EEA and TCA. The circle connecting the neural foramens was considered to be the border of EEA and TCA. The resected area by EEA and TCA covered all parts of the skull base.

### 4.2. Our Strategy for SBMs

Our strategy for SBMs has been to radically resect tumors with functional preservation. Several types of TCA for SBMs based on microsurgical anatomies have previously been reported [5,13,14,15,16,17,18,19,20]. In addition, a report on a series of 161 consecutive cases of SBMs who underwent TCA suggests that radical resection could lead to excellent tumor control [8]. We initiated the use of EEA, which had been used for pituitary adenomas, for SBMs in 2014. It was first adapted to parasellar meningiomas, which are located in a similar anatomical area as the pituitary adenomas. Therefore, the indications of EEA for SBMs have been expanding with the development of novel endoscopic surgical techniques and instruments. CSF leakage caused by extended surgical corridors is a common complication of EEA due to the difficulty in repairing the dura mater. Shannon et al. suggested that CSF leakage often occurs in anterior or posterior fossa meningiomas, and pedicle mucosal flaps could reduce the risk [21]. The cohort in this study included many patients with a high risk of CSF leakage. We routinely used pedicle mucosal flaps, accompanied by abdominal autologous fat for reconstruction. Although 15% of patients needed surgical repair after EEA, all the patients recovered.

### 4.3. Voxel-Based-Lesion Mapping for Skull Base Meningiomas

Previous reports regarding the extent of tumor resection were based on microscopic findings during the surgery [5,19,22,23]. However, this evaluation is challenging because it is difficult to recognize the resected areas objectively. Voxel-based-lesion mapping is quantitative and can be easily applied to every patient with SBMs. Surgeons can predict the resectable area by either of the approaches based on the previous outcomes and discuss the surgical strategies with patients as well as colleagues.

### 4.4. Limitations of This Study

The location, residual, and resected maps in the present study were created from our institutional data and may not be applicable to data from other institutions. Other limitations of the voxel-based-lesion mapping used in this study are as follows: VOIs were manually created, the number of EEAs was small, and enhanced lesions might include structures other than the remaining tumors. Future investigations should be performed with a large number of cases using automatic VOI creation.

## 5. Conclusions

Voxel-based-lesion mapping was used to visualize the resected area of SBMs by EEA and TCA quantitatively. Central SBMs could be resected well using EEA. The circle connecting the neural foramens was the visible border between both approaches. The resected area by EEA and TCA covered all parts of the skull base. The results of the present study might indicate the appropriate approach for SBMs.

## Figures and Tables

**Table 1:** Characteristics of patients who underwent surgery for WHO grade 1 skull base meningiomas between January 2014 and December 2019.

	EEA	TCA
**No. of surgeries**	20	135
**Male (*No. (%)*)**	7 (35.0)	36 (26.7)
**Mean age at surgery (*yrs*)**	57.8 ± 11.8 SD	57.8 ± 12.2 SD
***Tumor location***		
**Frontal base (*No. (%)*)**	3 (15.0)	31 (23.0)
**Sphenoid-Middle fossa (*No. (%)*)**	10 (50.0)	36 (26.7)
**Petroclival (*No. (%)*)**	7 (35.0)	46 (34.1)
**Posterior fossa (*No. (%)*)**	0 (0.0)	22 (16.3)
***Microscopic tumor removal rate***		
**Gross total resection (*No. (%)*)**	0 (0.0)	60 (44.4)
**Subtotal resection (*No. (%)*)**	17 (85.0)	63 (46.7)
**Partial resection (*No. (%)*)**	3 (15.0)	12 (8.9)
***Recurrence rate* (*No. (%)*)**	5 (25.0)	21 (15.6)
***Adjuvant therapy for recurrence tumor***		
**Radiation therapy (*No. (%)*)**	3 (15.0)	15 (11.1)
**Surgery (*No. (%)*)**	4 (20.0)	10 (7.4)
***Clinical outcome (KPS scores)***		
**Improved (*No. (%)*)**	1 (5.0)	19 (14.1)
**No changed (*No. (%)*)**	19 (95.0)	104 (77.0)
**Deteriorated (*No. (%)*)**	0 (0.0)	12 (8.9)
***Permanent neurological deficit***		
**Anosmia (*No. (%)*)**	0 (0.0)	3 (2.2)
**Visual defect (*No. (%)*)**	0 (0.0)	3 (2.2)
**Diplopia (*No. (%)*)**	1 (5.0)	19 (14.1)
**Facial numbness (*No. (%)*)**	0 (0.0)	11 (8.1)
**Facial palsy (*No. (%)*)**	0 (0.0)	3 (2.2)
**Hearing loss (*No. (%)*)**	0 (0.0)	2 (1.5)
**Dysphagia (*No. (%)*)**	0 (0.0)	7 (5.2)
**Motor (*No. (%)*)**	0 (0.0)	5 (3.7)
**Ataxia (*No. (%)*)**	0 (0.0)	1 (0.7)
**Death (*No. (%)*)**	0 (0.0)	1 (0.7)
**CSF leakage (*No. (%)*)**	3 (15.0)	2 (1.5)

KPS: Karnofsky performance status, CSF: cerebrospinal fluid, SD: standard deviation.

## Data Availability

Not applicable.
